# Aprocitentan in hypertension management: clinical efficacy, safety, and future prospects

**DOI:** 10.1097/MS9.0000000000003028

**Published:** 2025-02-07

**Authors:** Rumaisa Riaz, Usaid Ahmed, Unaiza Naqi, Laiba Afaq, Ayesha Shaukat, Yalnaz Khan, Aymar Akilimali

**Affiliations:** a Internal Medicine, Dow University of Health Sciences (DUHS), Karachi, Pakistan; b Department of Research, Medical Research Circle (MedReC), Goma, DR Congo

**Keywords:** blood pressure, endothelin-1, endothelin receptor antagonists, essential hypertension, hypertension

## Abstract

Hypertension (HTN) is a prevalent medical condition characterized by systolic blood pressure ≥ 130 mm Hg and diastolic blood pressure ≥ 80 mm Hg. In 2010, the global prevalence of HTN was 31.1%, with higher rates in men and low- and middle-income countries (LMICs). The etiology of primary HTN involves neurohumoral, renal, metabolic, genetic, and environmental factors, with the kidneys playing a significant role in long-term blood pressure regulation. Endothelin-1 (ET-1), a potent vasoconstrictor, contributes to HTN by affecting salt–water balance and promoting vascular remodeling. HTN often presents without symptoms, leading to complications such as heart failure, stroke, and renal failure if untreated. Common treatment options include angiotensin-converting enzyme inhibitors, angiotensin-receptor blockers, calcium channel blockers, and diuretics. Aprocitentan, a novel dual endothelin receptor antagonist, has shown promise in reducing blood pressure in patients with resistant HTN. Clinical trials, including the Phase 3 PRECISION study, demonstrated its efficacy and long-term control. However, Aprocitentan’s use is associated with safety concerns, such as hepatotoxicity, fluid retention, and embryo-fetal toxicity, necessitating careful monitoring. Aprocitentan represents a significant advancement in HTN management, offering a new therapeutic option for patients with uncontrolled HTN, although vigilant monitoring and informed decision-making are essential to mitigate potential risks and ensure optimal outcomes.

## Introduction

Hypertension (HTN) is a clinically significant condition identified by an elevation in systolic blood pressure (SBP) to ≥130 mm Hg and/or diastolic blood pressure (DBP) to ≥80 mm Hg. Chronic increased arterial pressure is the hallmark of this condition^[1]^. Notable differences were observed between high-income countries (HICs) and low- and middle-income countries (LMICs). HICs had a lower prevalence of HTN (28.5%) compared to LMICs (31.5%). Among men, the lowest prevalence was in South Asia (26.4%) and the highest in Eastern Europe and Central Asia (39.0%). Among women, the lowest prevalence was in HICs (25.3%) and the highest in Sub-Saharan Africa (36.3%)^[1]^. In the United States, approximately half of adults (115 million) had HTN in 2017–2018, with higher rates seen among Black individuals (58.53%), men (54.46%), older and obese individuals (61.03%), and those with diabetes or chronic kidney disease^[2]^. The age-adjusted prevalence of HTN in the US was 44.7% from 2017 to 2020, showing a steady rise compared to previous decades^[3]^. This article focuses on Aprocitentan, an Food and Drug Administration (FDA)-approved drug, exploring its mechanism of action, efficacy, and future aspects in the treatment of HTN.

**Highlights**

- HTN significantly contributes to cardiovascular morbidity and mortality, disproportionately impacting adults in LMICs.
- ET-1 exacerbates HTN by binding to endothelin A and endothelin B receptors, inducing vasoconstriction and vascular remodeling.
- Symptoms associated with HTN encompass neurological, cardiovascular, and systemic manifestations such as headaches, dizziness, irritability, palpitations, visual disturbances, and chest heaviness.
- Aprocitentan has gained Food and Drug Administration approval as a novel therapeutic option for adults with resistant HTN.
- The PRECISION Phase 3 trial has confirmed Aprocitentan’s efficacy in reducing blood pressure, warranting further investigation into its long-term safety and application scope.

## Pathophysiology

The etiology of primary HTN remains uncertain, with the condition linked to various neurohumoral, renal, metabolic, genetic, racial, and environmental factors. This multifactorial nature complicates the understanding of its pathophysiology^[4]^. The kidneys play a critical role in long-term blood pressure regulation, and impaired renal pressure natriuresis is a common feature in chronic HTN. Several factors, including decreased nitric oxide production, increased reactive oxygen species, inflammatory cytokines, and endothelin, contribute to this impairment^[5]^. Endothelin-1 (ET-1) is a potent vasoconstrictor and growth factor, raising blood pressure and causing vascular changes. It impacts salt–water balance by affecting hormonal systems, further elevating blood pressure. This vasoconstrictive effect is mediated by binding to endothelin receptors (endothelin A, ETA and endothelin B, ETB) on smooth muscle cells in blood vessel walls, triggering calcium influx and muscle contraction^[6]^. Additionally, ET-1 impacts hormonal systems such as the renin–angiotensin–aldosterone system (RAAS) and vasopressin release, leading to fluid retention, increased blood volume, and further elevation in blood pressure. Overexpression of ET-1 is observed in specific hypertensive models, particularly in salt-dependent HTN^[7]^. The exact causes of decreased renal function in primary HTN are not fully understood. However, dietary factors and excessive weight gain play a significant role, as HTN is less common in non-obese individuals^[5]^. Other mechanisms contributing to HTN include genetics, hyperactivity of the sympathetic nervous system, excessive sodium intake, obesity, metabolic syndrome, insulin resistance, and environmental factors^[8]^.

HTN often presents without noticeable symptoms, which makes routine blood pressure checks essential. Some common symptoms include headaches, dizziness, mood disturbances, palpitations, impaired vision, and chest heaviness^[9]^. If left untreated, HTN can lead to serious complications, including ventricular hypertrophy, heart failure, stroke, renal failure, and retinopathy^[10]^. It is also the leading cause of cardiovascular disease and premature death worldwide^[1]^.

## Current treatment options for HTN

Multiple treatment options are considered for HTN, among which angiotensin-converting enzyme inhibitors, angiotensin-receptor blockers (ARBs), calcium channel blockers (CCBs), and thiazide-like diuretics are prominent as first-line options. Below is a detailed viewpoint on antihypertensive agents^[11]^.

## Hydrochlorothiazide

Thiazides like hydrochlorothiazide are key in HTN treatment, reducing mortality^[12]^. They work by inhibiting sodium chloride transport in the distal convoluted tubule, increasing sodium excretion in urine. This leads to reduced water and sodium movement into the interstitium, initially lowering blood pressure via natriuresis and sustaining the long-term effect through vasodilation. The onset of the impact is rapid, peaking at 4 hours and lasting up to 12 hours. Starting dosage is typically 12.5–25 mg daily, potentially increasing to 50 mg daily. Side effects include hypokalemia, hyponatremia, hypercalcemia, and hyperchloremic alkalosis. It can elevate fasting glucose levels, cause gout flares, and rarely ocular disturbances. Patients with cirrhosis require close monitoring due to the risks of hepatic encephalopathy and severe hyponatremia^[13]^.

## Chlorthalidone

Chlorthalidone blocks the sodium–chloride cotransporter in the kidney, increasing sodium and water excretion, which reduces blood pressure through short-term volume adjustments and long-term effects. It may improve endothelial function and decrease oxidative stress. Like hydrochlorothiazide, it enhances potassium and hydrogen ion secretion and boosts calcium reabsorption. Its prolonged action makes it preferable for patients with adherence challenges^[14]^. However, it presents side effects, including hypokalemia and hyperuricemia, with a higher risk of arrhythmias like torsades de pointes. Combining it with antiarrhythmic drugs requires close potassium monitoring. Additionally, it poses risks of renal disorders, acute renal failure, chronic kidney disease, and increased risk of type 2 diabetes mellitus due to potassium depletion or dehydration^[15]^.

## Enalapril

The RAAS regulates blood pressure, with imbalance leading to HTN. Renin, from kidneys, converts angiotensinogen into angiotensin II, while aldosterone, from adrenal glands, enhances sodium reabsorption^[11]^. Enalapril, an ACE inhibitor, blocks RAAS by inhibiting angiotensin-converting enzyme, reducing angiotensin II levels. This lowers blood pressure and increases salt excretion^[16]^. However, enalapril can cause side effects like dry cough and angioedema due to bradykinin accumulation, leading to vasodilation. Approximately 20% of angioedema cases can be life-threatening, affecting the upper respiratory tract^[17]^.

## Atenolol

Atenolol is one of the many beta blockers that primarily works by blocking the action of catecholamines on beta-adrenergic receptors^[18]^. It mainly lowers blood pressure by decreasing cardiac output. However, this process may cause compensatory peripheral vasoconstriction, which could increase peripheral resistance and have a negative impact on the metabolism of fats and carbohydrates. When compared to other antihypertensive drugs, these side effects worsen the development of endothelial dysfunction and can cause new-onset diabetes, decreasing the medicines’ ability to lower the risk of stroke^[19]^.

## Clonidine

As a centrally acting alpha-2 agonist, this drug lowers blood pressure by inhibiting the release of norepinephrine. Due to its mode of action, it has many side effects (ranging from more serious central nervous system side effects like sleepiness and sedation to peripherally mediated constipation and dry mouth). As a result, it is typically saved for last-minute decisions. Additionally, a sudden increase in blood pressure is linked to tachycardia and severe rebound HTN; thus, a very progressive taper is needed to stop^[20]^.

## Hydralazine

The antihypertensive action of hydralazine is achieved through direct vascular dilatation. These medications must be used with a beta-blocker and diuretic to prevent the compensatory reflex tachycardia and water retention that ensue. Furthermore, as hydralazine is linked to an increased risk of drug-induced lupus, particular adverse effects should also be taken into account^[20]^.

## Angiotensin-receptor blockers

**Azilsartan medoxomil**, an ARB, inhibits angiotensin II type 1 (AT1) receptors in the RAAS, causing vasodilation and lowering blood pressure. Common side effects include dizziness, hypotension, gastrointestinal issues, muscle weakness, and electrolyte imbalances (elevated potassium, sodium, and uric acid). Coughing, hematologic abnormalities, and teratogenic effects are less frequent but notable. Azilsartan is contraindicated in pregnancy due to its potential to cause birth defects. Women of childbearing age require careful counseling and monitoring. Monitoring electrolytes and blood pressure is essential during treatment to manage side effects and ensure safety^[21]^.

**Olmesartan medoxomil** is a synthetic imidazole derivative ARB and antagonizes both, angiotensin type I and II receptors^[22]^, causing vasodilation, reduced aldosterone release, and arteriolar relaxation. This decreases sodium reabsorption, promoting diuresis (increased production of urine) and lowering blood pressure. Olmesartan is generally well-tolerated but can cause headaches, dizziness, and respiratory infections. Less common side effects include hyperglycemia, diarrhea, and elevated liver enzymes. Rare adverse effects like acute renal failure, alopecia, and enteropathy may occur. Olmesartan is contraindicated with ACE inhibitors and in pregnancy^[23]^. Long-term use can lead to sprue-like enteropathy symptoms, requiring careful monitoring for severe gastrointestinal issues^[23]^.

## Calcium channel blockers

**Nifedipine**, a dihydropyridine CCB, treats HTN and angina by blocking L-type calcium channels in cardiac and smooth muscle cells, reducing vascular resistance and improving coronary artery dilation. This action lowers blood pressure and enhances myocardial oxygen supply. Common side effects (20–30% incidence) include headache, dizziness, flushing, and peripheral edema due to vasodilation. Abrupt cessation may cause rebound HTN or angina. Immediate-release forms are risky for hypertensive emergencies. It is contraindicated in unstable angina without beta-blockade and hepatic impairment due to potential toxicity. Hypersensitivity reactions like pruritus and urticaria can occur. Extended-release forms offer better tolerance compared to immediate-release forms^[24]^.

**Amlodipine**, a long-acting dihydropyridine CCB, has a similar mechanism of action to Nifedipine, i.e., it works by blocking vascular L-type calcium channel^[25]^, resulting in vasodilation. Its prolonged half-life and high bioavailability make it effective for 24-hour blood pressure control with a single dose. Amlodipine is well-tolerated in diabetes and kidney disease patients, maintaining renal and glycemic function. Common side effects include palpitations, flushing, edema, and dizziness, more prominent at higher doses. Gum hypertrophy, occurring in about 2% of cases, is reversible upon discontinuation. Amlodipine benefits elderly patients, offering blood pressure regulation and cardiovascular protection against myocardial infarction and stroke^[26]^.

## Endothelin receptor antagonists

The endothelin pathway is involved in the crucial vasoconstrictive action to regulate the vascular tone and blood pressure. Endothelins are potent vasoconstrictor peptides with ET-1 found in the cardiovascular system, while other peptide isoforms are ET-2 and ET-3. These peptides exert their effect by binding to endothelin receptors at smooth muscles cells. The excessive production of endothelin in the lung can cause pulmonary arterial hypertension (PAH). This is treated with endothelin receptor antagonists (ERAs) such as bosentan, ambrisentan, and macitentan which mitigate PAH development and relieves the symptoms. Bosentan, however, is a non-peptide pyrimidine derivative which was the first ERA to be approved for PAH treatment in patients with a World Health Organization (WHO) functional classes III–IV^[27]^. A long term, double randomized controlled trial called SERAPHIN evaluated the safety and efficacy of macitentan 10 mg in 742 patients. The most common side effects were peripheral edema, upper respiratory tract infection, and nasopharyngitis, an increase in ALT or AST^[28]^. More importantly, a recent meta-analysis was conducted in which safety and efficacy of the transition from ambrisentan and bosentan to macitentan was evaluated, it showed that the latter has a better safety profile and benefits than the former two drugs, with better results in 6-minute walk test and WHO functional class as well as better liver function than with bosentan. But the side effects of ERAs are still quite a lot in number and abovementioned adverse effects like peripheral edema as well as the addition of liver dysfunction, headaches, menstrual irregularities, and anemia were a significant concern as well^[29]^. The REPAIR study also noted the same adverse effects as mentioned above and one death occurred as well as due to a fatal significant adverse event cardiac arrest^[30]^.

## Aprocitentan as drug therapy

**Figure 1.**
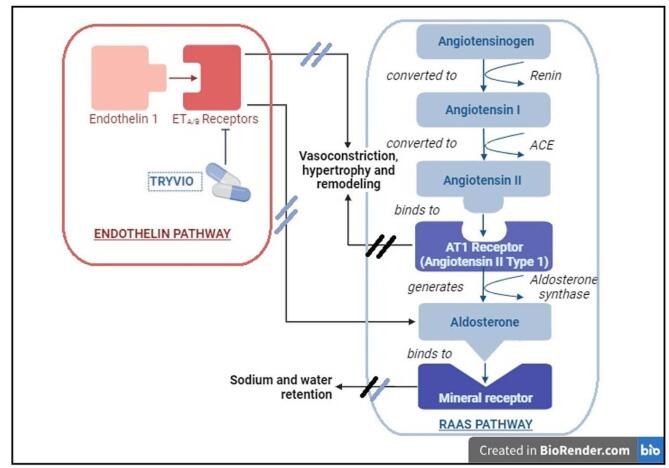
Mechanism of action of aprocitentan: modulation of endothelin and RAAS pathways.

The FDA has recently granted approval for Aprocitentan (marketed as Tryvio) to be used alongside other antihypertensive medications in adults with uncontrolled HTN, aiming to reduce their blood pressure levels^[31]^. Aprocitentan is a highly effective oral medication that functions as an antagonist for both ETA and ETB receptors, with a potency ratio of inhibiting ETA to ETB receptors at 1:16^[31]^ It mitigates negative effects of ET-1, such as blood vessel constriction and tissue scarring. In conditions like HTN, ET-1 can lead to vessel dysfunction and increased sympathetic activity^[32]^. This mechanism works in parallel with the RAAS pathway, where angiotensin II typically induces similar effects through the AT1 receptors, ultimately promoting aldosterone production and sodium and water retention. By intervening in these pathways, Tryvio effectively lowers blood pressure and counters the mechanisms that contribute to HTN (Figure 1). In contrast to Bosentan but similar to Macitentan, Aprocitentan does not disrupt bile salt balance and does not induce liver damage^[33]^.

ETA/B receptors, endothelin A (ETA) and endothelin B (ETB) receptors; ACE, angiotensin-converting enzyme; AT1 receptor, angiotensin II type 1 receptor; RAAS, renin–angiotensin–aldosterone system *(created with biorender.com).*

## Clinical efficacy of aprocitentan

Aprocitentan underwent evaluation as a standalone treatment in a Phase 2 trial involving patients with HTN^[34]^ and as an add-on therapy in the Phase 3 PRECISION study, which focused on patients with confirmed resistant HTN^[35]^.

### Insights from the FDA-approved PRECISION trial

The FDA has granted approval for Aprocitentan based on results from the Phase III PRECISION trial (NCT03541174), a multicenter, blinded, randomized, parallel-group study involving adults with SBP ≥ 140 mmHg despite treatment with three or more antihypertensive medications (Table 1). This multicenter trial, comprising 730 patients with resistant HTN, assessed the short- and long-term efficacy of Aprocitentan, a combination therapy including an ARB, a CCB, and a diuretic^[32,35]^.

**Table 1:** Summary of the PRECISION and HTN trial.

Study ID	Drug	Phase	Sample size	Outcomes	Adverse events	References
PRECISION trial (NCT03541174)	Aprocitentan (12.5 and 25 mg) with phased placebo and re-randomization phases	Phase 3	730 patients with resistant HTN (SBP ≥140 bpm) despite treatment with ≥3 antihypertensive drugs	Aprocitentan 12.5 mg was more effective than placebo in reducing sitting SBP at Week 4 with sustained effect.	Edema/fluid retention (9.1%), anemia (3.7%), minor hypersensitivity (0.8%), slight initial eGFR decline, and hemoglobin reduction	^[29,32]^
HTN trial (NCT02603809)	Aprocitentan (5, 10, 15, and 25 mg) compared with placebo and lisinopril (20 mg)	Phase 2	930 patients in single-blind placebo run-in; 490 randomized with DBP 90–109 mmHg, 430 completed the treatment	Aprocitentan doses (10, 25, and 50 mg) showed significant reduction in sitting systolic/diastolic automated office BP compared to placebo or lisinopril from baseline to Week 8	Common adverse events: headache, HTN, nasopharyngitis; mild peripheral edema; reduction in hemoglobin, hematocrit, and albumin	^[31]^

HTN, hypertension; SBP, systolic blood pressure; eGFR, estimated glomerular filtration rate; bpm, beats per minute; DBP, diastolic blood pressure; BP, blood pressure.

The trial was structured into three phases: a 4-week double-blind phase comparing Aprocitentan (12.5 mg or 25 mg) to placebo, followed by a 32-week single-blind phase with Aprocitentan 25 mg, and concluding with a 12-week randomized withdrawal phase with Aprocitentan 25 mg or placebo^[32,35]^.

The main goal of the study was to see how much sitting systolic blood pressure (SiSBP) could be reduced from the start of the study to Week 4. This was measured using an unattended automated office blood pressure (uAOBP) device during the initial phase. The results showed that a 12.5 mg dose of Aprocitentan lowered SiSBP much more effectively than a placebo by Week 4. The study also looked at how well Aprocitentan worked overtime. During the withdrawal phase, patients who were switched back to a 25 mg dose of Aprocitentan continued to have better blood pressure control than those who were switched to a placebo. These results highlight that Aprocitentan is a strong option for treating patients with resistant HTN, as it provides consistent blood pressure control over the long term.^[32,35]^

### *The Phase 2 trial: assessing Aprocitentan’s response in essential* HTN

The Phase 2 HTN trials evaluated the impact of Aprocitentan on essential HTN. In this randomized, double-blind study, participants with DBP between 90 and 109 mmHg received varying Aprocitentan, placebo, or lisinopril over 8 weeks (Table 1). Aprocitentan, known for its dual ETA/ETB receptor antagonist properties, was investigated for its effectiveness. Automated office blood pressure measurements were taken at various intervals, while ambulatory blood pressure was monitored over 24 hours. Following a single-blind placebo run-in phase, 490 individuals advanced to the double-blind phase, with 409 completing the therapy as per protocol. Results indicated significant reductions in sitting systolic/diastolic uAOBP with Aprocitentan, particularly at 10, 25, and 50 mg doses, compared to placebo and lisinopril. Additionally, Aprocitentan significantly reduced 24-hour blood pressure among patients with valid ambulatory data^[34]^.

## Safety profile of Aprocitentan

The safety of Aprocitentan was assessed in a placebo-controlled Phase 3 study which included 724 patients with varying treatment durations. The most reported adverse reactions were edema/fluid retention and anemia with a frequency of 9.1% and 3.7%, respectively. Hypersensitivity reactions were rare, with 0.8% of Aprocitentan-treated patients experiencing them compared to none in the placebo group. Due to the diverse conditions in clinical trials, the rates of adverse reactions observed in one drug’s trials cannot be directly compared to those of another drug^[32,36]^. Additionally, the initiation of Aprocitentan might lead to a slight initial decrease in estimated glomerular filtration rate within the first 6 weeks of therapy, followed by stabilization. During the first 4 weeks of the double-blind treatment, Aprocitentan 12.5 mg caused an average reduction of approximately 0.8 g/dl in hemoglobin levels, contrasting with no alteration observed in the placebo recipients^[32,36]^. There were no notable differences in how Aprocitentan behaved in the body across different demographics, health conditions, and liver or kidney function levels, except for severe liver or kidney impairment where its effects are still uncertain.

Aprocitentan has a manageable safety profile with no significant drug–drug interactions and is generally easy to prescribe and use, making it a convenient option for many patients. Considering the points discussed above, it is advisable to avoid Aprocitentan in specific patient groups. It is not yet known whether Aprocitentan passes into breast milk, so breastfeeding is not recommended while taking this medication. For those breastfeeding or planning to breastfeed, it is important to discuss the best feeding options for the baby with a health care provider. Additionally, Aprocitentan may cause serious side effects, including severe birth defects if taken during pregnancy. Therefore, people who can become pregnant must ensure they are not pregnant before starting treatment with Aprocitentan, avoid becoming pregnant during treatment, and for 1 month after stopping the medication. Second, individuals at risk for hepatotoxicity should be informed about the signs of liver toxicity and encouraged to promptly consult their health care provider if any symptoms manifest. Finally, patients should be vigilant regarding fluid retention, and any instances of significant weight gain or excessive swelling in the lower extremities should be promptly assessed by a health care professional.

## Future applications and limitations

HTN presents a significant public health concern, heightening the risk of cardiovascular and cerebrovascular events. Despite available treatments, uncontrolled HTN persists among many patients. The endothelin pathway, a crucial contributor to HTN, was largely overlooked until the development of Aprocitentan^[37]^. Aprocitentan, an ERA, effectively targets ET-1 binding, marking a pioneering approach in HTN management. Aprocitentan’s approval signifies a paradigm shift, the first oral antihypertensive in nearly four decades, promising transformative progress in systemic HTN treatment. Its once-daily administration complements existing therapies without inducing drug interactions, ensuring ease for physicians and patients^[36]^.

Moreover, its consistent efficacy across diverse patient demographics and clinical profiles underscores its potential to revolutionize HTN management. Pharmacokinetically, Aprocitentan exhibits favorable characteristics, including a prolonged half-life and minimal clearance, and is unaffected by dietary intake, ensuring consistent therapeutic efficacy. These features highlight Aprocitentan’s potential to enhance patient outcomes and alleviate the burden of cardiovascular disease^[32]^.

Despite its promising therapeutic potential, the journey of Aprocitentan is accompanied by notable challenges and safety considerations. Chief among these concerns is the potential seriousness of its adverse effects, necessitating the inclusion of a boxed warning to caution health care practitioners. Particularly significant are the risks associated with hepatotoxicity and liver failure, prompting the recommendation for monitoring serum aminotransferase levels and total bilirubin both before initiating treatment and periodically after that, as clinically indicated. In addition to hepatic concerns, Aprocitentan usage has been associated with various other adverse effects, including fluid retention, reductions in hemoglobin levels, decreased sperm counts, and the potential for embryo-fetal toxicity^[32]^. These safety considerations underscore the importance of vigilant monitoring and informed decision-making when prescribing Aprocitentan, highlighting the need for close collaboration between health care providers and patients to mitigate potential risks and ensure optimal therapeutic outcomes.

## Conclusion

HTN remains a prevalent and severe public health concern worldwide, significantly increasing the risk of cardiovascular and cerebrovascular events. Despite the availability of numerous antihypertensive therapies, effective management remains challenging, particularly in patients with resistant HTN. Aprocitentan represents a significant advancement in this field, offering a novel therapeutic option that sets it apart from existing treatments. Unlike conventional antihypertensive agents targeting pathways such as the RAAS or calcium channels, Aprocitentan operates through a unique dual antagonism of both ETA and ETB receptors. This mechanism disrupts the endothelin pathway, a critical contributor to vascular dysfunction and HTN, thereby reducing vasoconstriction and mitigating tissue fibrosis and inflammation – factors often exacerbated in patients with resistant HTN. Clinical trials, including the pivotal Phase 2 and Phase 3 PRECISION studies, have demonstrated Aprocitentan’s efficacy in significantly lowering SBP and maintaining long-term control, particularly in patients who have not responded adequately to traditional therapies. Furthermore, Aprocitentan has shown a favorable safety profile, avoiding the hepatotoxicity commonly associated with earlier ERAs and maintaining bile salt balance, which enhances its suitability for long-term use. However, the use of Aprocitentan is not without safety considerations. Potential risks, including fluid retention, hepatotoxicity, and embryo-fetal toxicity, require vigilant monitoring by health care providers to ensure safe and effective use. Despite these challenges, Aprocitentan’s dual-action approach and its ability to address unmet needs in HTN management make it a promising new addition to the therapeutic arsenal against this widespread condition.

## Data Availability

Not available.
